# The trend of radiological severity of hip fractures over a 30 years period: a cohort study

**DOI:** 10.1186/s12891-019-2739-1

**Published:** 2019-08-07

**Authors:** Mehdy Farhang, Sebastian Mukka, Ulrica Bergström, Olle Svensson, Arkan S. Sayed-Noor

**Affiliations:** Department of Surgical and Perioperative Sciences, Umeå University, 90187 Umeå, Sweden

**Keywords:** Hip fracture, Proximal femoral fracture, Trend, Severity

## Abstract

**Background:**

Despite advances in operative techniques and preoperative care, proximal femur fractures (PFF) still represent a great public health problem. Displacement and fracture stability have been assumed as important determinants of treatment modality and outcome in such fractures. Purpose of this study was to determine whether the radiological severity of PFF fractures has increased over time.

**Methods:**

In a cohort study, the plain radiographs of all patients with PFF aged over 50 years who were admitted to Umeå University Hospital in 1981/82, 2002 and 2012 were recruited to examine the types of fractures.

**Results:**

The ratio of undisplaced to displaced femoral neck (FN) fractures was 30 to 70% in 1981/82, 28 to 72% in 2002 and 25 to 75% in 2012. The ratio of stable to unstable intertrochanteric (IT) fractures was 64 to 36% in 1981/82, 68 to 32% in 2002 and 75 to 25% in 2012. The ratio of simple to comminute subtrochanteric fractures was 35 to 65% in 1981/82, 16 to 84% in 2002 and 12 to 88% in 2012. In both FN and IT fractures we found no statistical difference among these 3 study periods, *p* = 0.67 and *p* = 0.40. In subtrochanteric fractures we saw a tendency towards more comminute subtrochanteric fractures (1981/82 to 2012), *p* = 0.09.

**Conclusions:**

We found no significant increment in the radiological severity of FN and IT over a 30 years’ period. However, there was tendency towards an increase in comminute subtrochanteric fractures.

## Background

Osteoporotic fractures and mainly those involving the proximal femur (PFF) represent a great public health problem, with a drastic economic burden on the community. In USA, nearly 300,000 PFF occur every year at a cost of over 8.8 billions USD while in Sweden, nearly 18,000 PFF occur every year at a cost of 200 millions USD [1–3]. Fractures of the femoral neck (FN) and the intertrochanteric (IT) region account for more than 90% of PFF and occur in approximately equal proportions. The incidence of these fractures seems to be flattening despite previous reports indicating a trend of increasing incidence as the population ages [4]. The mechanism of injury and risk factors for FN fractures differ from those associated with IT fractures and the FN fractures are more prevalent in people with impaired functional status and corticosteroid use while IT fractures occur in older and thinner patients with poor health status [5].

The functional outcome of PFF can be determined by several factors with variable influence. These factors include age, gender, preoperative morbidities, pre-fracture residence and ambulation, fracture type and functional status at hospital discharge [6, 7]. Many of these patients never regain their pre-fracture walking ability and social independency. It is generally accepted that the displacement of FN fractures and the stability of IT fractures are important determinant of the treatment modality and outcome.

The purpose of this study was to review the radiological types of PFF treated at a single university hospital over a 30 years’ period, to determine whether the severity of these fractures has increased with time. Our null hypothesis was that no such severity increment existed.

## Methods

### Study setting

Umeå University Hospital is situated in the Northern part of Sweden and is responsible for all the emergency care for the city of Umeå and its surrounding neighbourhoods (145,000 inhabitants and 10,730 km^2^). There is no other hospital in this part of Västerbotten County.

In retrospective cohort study, all patients with PFF (FN, IT and subtrochanteric fractures) aged over 50 years who were admitted to Umeå University Hospital during years 1980–2014 were included. Pathologic and peri-prosthetic fractures were excluded. The unique Swedish personal identification numbers were used.

### Data collection

The database (UmanHip database) was compared with the hospital registers on an annual basis; thus, all in-hospital fractures were also registered. By crosschecking against the hospital’s compulsory e-code registration regarding the reason for hospital admission, any possibility of losing in-patients in the data set was minimized. The following variables were registered: age, gender, side of fracture, fracture type (FN, IT or subtrochanteric, without grading), operation date, operative technique and reoperations.

The plain radiographs (antero-posterior and lateral views) of all PFF patients from 1981/82, 2002 and 2012 were recruited to examine the types of fracture. The FN fractures were classified as undisplaced (Garden 1 and 2 types) or displaced (Garden 3 and 4 types) [8], while the IT fractures were classified according to Evan [9] as stable (Simple non-comminute fractures) or unstable (with comminution of the calcar or lateral cortex with fragment larger than 5 mm). Subtrochanteric fractures were defined as those whose main fracture line lay between the lesser trochanter and 5 cm distal to it, and classified according to Seinsheimer [10] as simple 2 fragments or comminute 3 or 4 fragments.

Radiographs from 1981/82 were available as analogue pictures while radiographs from 2002 and 2012 were available as digital pictures using PACS system.

To determine the interobserver and intraobserver reliability of the radiological measurements used in this study, a random set of digital radiographs (*n* = 244) were independently examined by two examiners, an orthopaedic surgeon and a radiologist, two times with 4–6 weeks’ interval. The remaining radiographs (*n* = 412) were examined and classified by the orthopaedic surgeon.

### Statistical analysis

The ages of the patients were compared using one-way analysis of variance (ANOVA) with post-hoc analysis. The distribution of gender and type of fracture was analysed with chi-squared test. Interobserver and intraobserver reliability was studied using Cohen’s Kappa Coefficient. We considered agreement as poor if <0.2, fair if between 0.21 and 0.40, moderate if between 0.41 and 0.60, substantial if between 0.61 and 0.80 and good if > 0.80. A *p*-value < 0.05 was considered to be statistically significant. Statistical analyses were performed using SPSS 17.0 (IBM, Armonk, New York).

## Results

There were 7737 PFF [4342 FN fractures (56%), 2772 IT fractures (36%) and 623 subtrochanteric fractures (8%)] admitted to Umeå University Hospital during the study period [nearly 30% were men and 70% were women, mean age of 81 (SD 9)]. The age-adjusted incidence per 100,000 and year was 799 in 1981/82, 637 in 2002 and 515 in 2012.

The Cohen’s Kappa coefficient for interobserver reliability was 0.85 (95% confidence interval 0.79–0.89) and for the intraobserver reliability was 0.89 (95% confidence interval 0.84–0.93).

There were 363 PFF fractures from 1981/82, of which analogue radiographs of 211 (58%) PFF were available. There were 229 PFF fractures from 2002, of which digital radiographs of 218 (95%) PFF were available while there were 235 PFF fractures from 2012, of which digital radiographs of 227 (97%) PFF were available.

### The trend of radiological severity of PFF fractures

In 1981/82, the number of undisplaced FN fractures was 36 (30%) compared to 85 (70%) displaced FN fractures. In 2002 the number of undisplaced FN fractures was 33 (28%) compared to 86 (72%) displaced FN fractures, while in 2012 the number of undisplaced FN fractures was 28 (25%) compared to 86 (75%) displaced FN fractures. We found no statistical difference among these 3 study periods, *p* = 0.67 (Table 1).

**Table 1:** Results of the trend of radiological severity of hip fractures showing no differences over the study period

	1981/82	2001	2012	***p***-value for difference among the 3 time periods
Available radiographs	211	218	227	
Undisplaced FN fractures	36 (30%)	33 (28%)	28 (25%)	*p* = 0.67
Displaced FN fractures	85 (70%)	86 (72%)	86 (75%)	
Stable IT fractures	47 (64%)	38 (68%)	53 (75%)	*p* = 0.40
Unstable IT fractures	26 (36%)	18 (32%)	18 (25%)	
Simple subtrochanteric fractures	6 (35%)	7 (16%)	5 (12%)	*p* = 0.09
Comminuted subtrochanteric fractures	11 (65%)	36 (84%)	37 (88%)	

In 1981/82, the number of stable IT fractures was 47 (64%) compared to 26 (36%) unstable IT fractures. In 2002 the number of stable IT fractures was 38 (68%) compared to 18 (32%) unstable IT fractures, while in 2012 the number of stable IT fractures was 53 (75%) compared to 18 (25%) unstable IT fractures. We found no statistical difference among these 3 study periods, *p* = 0.40 (Table 1).

In 1981/82, the number of simple subtrochanteric fractures was 6 (35%) compared to 11 (65%) comminute subtrochanteric fractures. In 2002, the number of simple subtrochanteric fractures was 7 (16%) compared to 36 (84%) comminute subtrochanteric fractures while in 2012, the number of simple subtrochanteric fractures was 5 (12%) compared to 37 (88%) comminute subtrochanteric fractures. There were a tendency for statistical difference among these 3 study periods, *p* = 0.09 (Table 1).

## Discussion

In this retrospective study, we found no significant increment in the radiological severity of FN and IT over a 30 years’ period. However, there was a tendency towards an increase in comminute subtrochanteric fractures.

The bone composition of the proximal femur differs between the FN, IT and subtrochanteric regions and therefore it is possible that the etiology of the fractures in these different sites may also differ. The trochanteric region for instance has a greater proportion of trabecular bone compared with the FN and subtrochanteric regions [11]. Also, the geometric parameters such buckling ratio and hip axis length were more strongly linked with the severity type of PFF, although interaction terms were mostly not significant [12]. Worldwide, the recent trends in the incidence of PFF have varied widely: increase, plateau, or decrease. Epidemiological studies have shown that the incidence increases gradually with age, starting at 40 years, with a steep increase after 75 years of age [4, 13, 14]. The main underlying etiology is low bone mineral density (BMD) and increasing risk for falling.

Cauley et al. [5] studied the risk factors affecting the degree of severity of FN and IT fractures, determined by the degree of fracture displacement using the Garden’s classification in FN fractures and the Kyle system for IT fractures, and found that displaced FN fractures were more common in older age, lower BMD, taller stature, corticosteroid use and poor functional status as measured by lower grip strength. On the other hand, Parkinson’s disease and poor vision were associated with stable IT fractures. Chehade et al. [15] for instance found that unstable IT fractures were at greater odds of postoperative complications, reoperation and mortality within 6 and 12 months than those with stable fractures. On the other hand, Cornwell et al. [16] compared the functional outcomes and mortality among patients with different types of PFF classified as either non-displaced FN, displaced FN, or stable IT and unstable IT fractures. Despite that mortality was highest for displaced FN fracture patients and functional independence measure scores were least for unstable IT fracture patients compared with non-displaced FN fracture patients respectively, a multivariate analysis identified pre-injury age and function as predictors for mortality and functional outcome.

The results of this study concur with those reported by Lakstein et al. who also found no differences between the radiological severity of FN and IT fractures between 2001 and 2010 [17]. Only the IT fractures in patients older than 80 years of age showed increased proportion of unstable fractures. Contrary to this, Martínez et al. [18] found a significant increase in the incidence of displaced FN fractures and a decrease in the incidence of undisplaced FN fractures in women, while the incidence of different types of trochanteric fractures did not vary. Regarding subtrochanteric fractures, our results showed increased proportion and severity of comminuted fractures during the study period. The annual report of Swedish National Registry of hip fracture patient care in 2016 demonstrated a gradually increasing incidence of displaced FN and unstable IT fractures during the last three decades, both for the entire country and the geographical area of the present study population [19]. The treatment methods of these fractures have also changed towards the use of hip arthroplasty and intramedullary nail rather than screw fixation and sliding screw and plating, respectively. The report however does not include analyses whether these incidence and treatment method changes reach statistical significance. The variation of results reported in these studies is probably related to the geographical, time- and population-related factors affecting the fracture severity e.g. BMD, body mass index, medications and co-morbidities. In the present study, for instance, the mean age at hip fracture increased from 76.5 years in early 80s to 81.5 years in 2012. This could explain the tendency towards more comminuted fractures in older osteoporotic patients in later years. This is in contrast to the increasing consumption of anti-resorptive medications and corticosteroids in recent years. Other possible influencing factors include changing in injury panorama from high to low energy trauma and falls, smoking and alcohol habit. As the present study is a radiological analysis we have not included these factors in our evaluation. On the other side, there is no obvious reason for the increased comminution of subtrochanteric fractures in comparison to the IT counterparts. However, a gradually increasing lifespan, an age dependent inactivity and decrease in BMD might contribute to this finding.

This study has a number of limitations. First, it is a radiological analysis with no clinical parameters included. Factors like pre-fracture BMD, postoperative hospital stay length, complications and reoperations and mortality can reflect the fracture severity. Second, approximately 40% of 1981/82 radiographs were unavailable for analysis. However, we think this absence is random with no influence on the validity of the study results, but a selection bias cannot be ruled out which could explain the differences found in subtrochanteric fractures. Radiographs from 1981/82 were available as analogue form and therefore could not be blinded. This might have influenced the observers. However, the inter- and intraobserver reliability were very good. Third, our sample size is limited and we are not able to detect smaller differences in fracture pattern. Furthermore, the results can only be generalized to populations similar to those included in this study i.e. white elderly patients living in comparable socio-economic and environmental circumstances. These limitations are counterbalanced by the strengths of the study, which covers 30 years’ data derived from a valid database on a stable population within a catchment area of a single center.

## Conclusions

We found no significant increment of radiological fracture severity of FN and IT fractures over the study period while the subtrochanteric fractures showed a tendency for increased complexity with time. These results can assist caregivers to predict and plan the resources needed to manage these important fractures in the elderly population.

## Data Availability

All data is stored in the trial registry. And the datasets used or analysed during the current study are available from the corresponding author on reasonable request.

## Abbreviations

ANOVA: Analysis of variance
FN: Femoral neck
IT: Intertrochanteric
PACS: Picture archiving and communication system
PFF: Proximal femur fracture
